# Study of Light-Activated Regioregular Poly(3-Hexyltiophene) Photoconductive Polymer Sensing Properties in Nerve Agent Simulant (DMMP) Detection^†^

**DOI:** 10.3390/s20020491

**Published:** 2020-01-15

**Authors:** Paulina Powroznik, Wiesław Jakubik, Agnieszka Stolarczyk, Anna Kazmierczak-Balata, Jaroslaw Wrotniak, Tomasz Jarosz

**Affiliations:** 1 Institute of Physics CSE, Silesian University of Technology, 44-100 Gliwice, Poland; wieslaw.jakubik@polsl.pl (W.J.); anna.kazmierczak-balata@polsl.pl (A.K.-B.); 2 Department of Physical Chemistry and Technology of Polymers, Silesian University of Technology, 44-100 Gliwice, Poland; agnieszka.stolarczyk@polsl.pl; 3 Institute of Electronics, Silesian University of Technology, 44-100 Gliwice, Poland; jaroslaw.wrotniak@polsl.pl

**Keywords:** regioregular poly(3-hexyltiophene), nerve agents sensor, sarin detection, DMMP, light activation, room temperature

## Abstract

In the present work, we report the use of regioregular poly(3-hexyltiophene) polymer (RR-P3HT) as a potential light-activated material for sensing the chemical nerve agent simulant dimethyl methylphosphonate (DMMP). The electrical response of thick films of RR-P3HT, deposited by spray-coating method onto a porous laminate substrate at room temperature, to DMMP vapours was investigated. The studied material was activated by light-emitting diodes that emitted light of different wavelengths. The sensing properties of RR-P3HT are considerably enhanced upon exposure to blue and yellow light. However, excitation by the low wavelength light (blue) caused degeneration of the material, resulting in lowered stability. In the case of the yellow light, degeneration was much slower and the limit of detection was 0.4 ppm. The studied material exhibited high selectivity, as it did not respond to 6 ppm of acetone and methanol vapours.

## 1. Introduction

Chemical warfare agents (CWA), especially nerve agents (e.g., sarin and soman), are highly lethal compounds. Sarin is one of the best-known chemical weapons of mass destruction. This odourless and colourless compound causes neuromuscular paralysis and death by suffocation within 1–10 min of exposure. Disabling and lethal exposures to sarin occur in 10 min above 15 and 64 ppb, respectively [1]. Since they can be used for military tests and against civilians, the development of fast CWA sensors with low detection thresholds is considered an urgent issue. Studies are usually carried out using dimethyl methylphosphate (DMMP), a non-toxic organophosphorus compound with a similar structure to sarin. In recent years, many materials like carbon nanotubes [2,3], zeolites [4] and organic compounds [3,5,6,7] have been investigated as DMMP sensors. In the field of gas sensors, growing attention has been paid to organic semiconductors in particular and many investigations of their properties as potential sensing materials have been reported [8,9,10,11,12,13]. The two primary advantages of organic gas sensors are their low work temperature and low power consumption [14].

Conductive polymers can be an effective medium for sensing organophosphates. Polyaniline, polysiloxane and poly(3-methyltiophene) (PMeT) [3,6,15,16,17] are all examples of such materials. Gas adsorption of DMMP vapours causes electronic changes in the thin films of these polymers. In the present study, we propose regioregular poly (3-hexyltiophene) (RR-P3HT) as a potential material that is sensitive to DMMP. This polymer is of particular interest because it exhibits a large hole mobility and strong photoconductivity [18]. Consequently, its sensing properties can be enhanced by the light [19]. In a previous work [20], we presented the preliminary results of our investigations of the capability of RR-P3HT—activated using white light—of detecting DMMP. In this study, we activated the RR-P3HT films by means of a variety of light-emitting diodes with a wide spectrum of wavelengths. The main reason for applying the light activation was due to the high photoconductivity of the studied polymer. The resistance of the deposited layer in dark conditions was on the order of G to T. For such a low dark conductance, measuring the sensing structure’s electrical response becomes complex, since it requires an adequate picoammeter and the appropriate screening of the setup. Since, in the previous study [20], we observed the sensitivity of RR-P3HT to DMMP while illuminated by a white light, in this work we focused on the investigation of the influence of the applied wavelength on sensing responses.

## 2. Materials and Methods

### 2.1. Synthesis of P3HT Polymer

The following were used as commercially available, without further purification: 2,5-dibromo-3-hexylthiophene (>97%) (TCI Chemicals), t-butylmagnesium chloride (2M diethyl ether solution) (Sigma Aldrich) and [1,3-bis (diphenylphosphino) propane] nickel (II) chloride (99%) (Acros Organics). Anhydrous tetrahydrofuran THF (99.9%) (Acros Organics) was distilled directly prior to use. All reactions were conducted under dry nitrogen flow, in oven-dried glassware.

**Figure A1:**
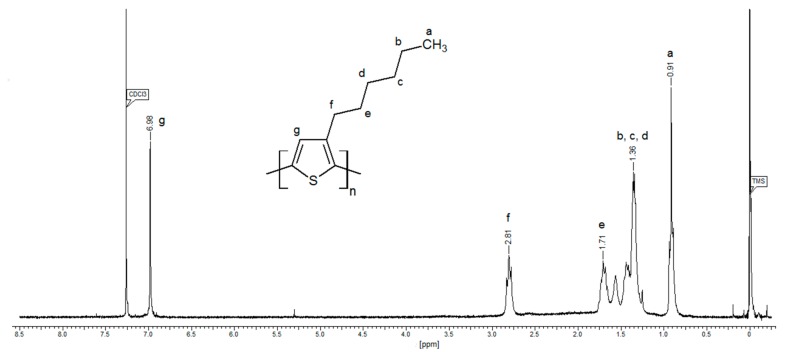
The ^1^H NMR spectrum of the synthesised P3HT sample.

To synthesise the hydrogen/bromine-terminated RR-P3HT, the McCullough GRIM method was used [21], with some modifications implemented, as per De Girolamo et al. [22]. A dry 100 mL three-necked flask, equipped with a septum, a condenser (end-capped with a trap to seal the reaction Lessel), a gas capillary and a magnetic dipole was flushed with nitrogen and charged via a syringe with 2,5-dibromo-3-hexylthiophene (6.13 mmol, 2 g), anhydrous THF (21.5 mL) and t-butylmagnesium chloride (7.46 mmol, 3.7 mL). The reaction mixture was refluxed for 2 h, followed by the addition of Ni (dppp) Cl2 catalyst (0.0318 mmol, 0.0172 g), then refluxed again for the next 1 h. The crude polymer was precipitated by quenching the reaction mixture in methanol and then it was filtered and purified by sequential Soxhlet extraction with methanol, hexane and chloroform. The polymer fraction was isolated from the extract (the fraction is soluble only in chloroform) as described above, yielding 0.411 g (yield 41%) of a dark green, brittle product. The chemical structure of the product was confirmed by proton nuclear magnetic resonance (1H-NMR) spectroscopy. NMR analyses were performed on solutions in CDCl_3_ on a Varian Unity Inova (USA) spectrometer with a resonance frequency of 300 MHz, using tetramethylsilane (TMS) as an internal standard: RR-PHT: 1H-NMR (CDCl3, 300 MHz) δH, ppm: 6.98 (s, 1H), 2.81 (m, 2H), 1.76–1.66 (m, 2H), 1.47–1.34 (m, 6H), 0.91 (t, J = 6,9 Hz, 3H). Sample 1H NMR spectrum is included as Figure A1.

**Figure A2:**
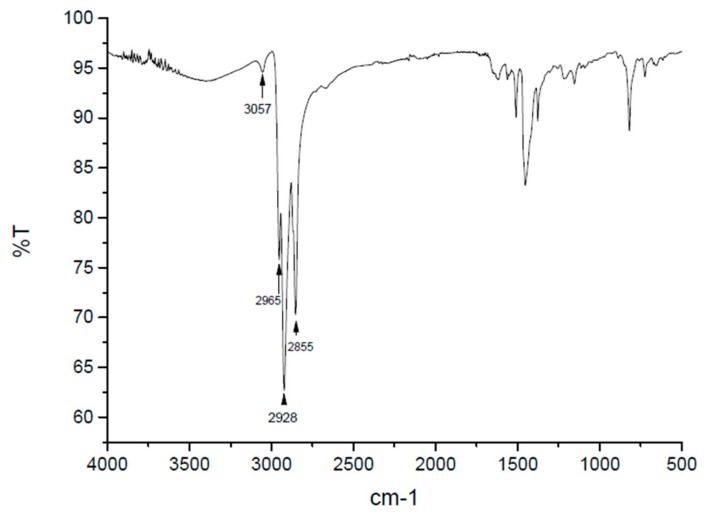
The FT IR spectrum of the synthesised P3HT sample.

IR spectra were acquired using a Perkin–Elmer Spectrum Two (Waltham, MA, USA). Signals were observed at: 3057 (ν C_Ar_–H w), 2965 (ν_asym_ C–H s), 2928 (ν_asym_ C–H s), 2855 (ν_sym_ C–H s), 1620 (ν C = C w), 1563 (ν C_Ar_ = C_Ar_ w), 1511 (ν_sym_ C_Ar_ = C_Ar_ m), 1456 (δ_asym_ C–H [–CH_3_] m), 1374 (δ_sym_ C–H [–CH_3_, –CH_2_–] m), 1217 (ν C_Ar_ = C_Ar_ w), 819 (δ C_Ar_–H m), 724 (δ = C–H; δ –(CH_2_)_n>3_ w), 668 (δ = C–H w). Sample IR spectrum is included as Figure A2.

### 2.2. Preparation of the Sensing Layer

The thick layer of RR-P3HT (with dimensions of 1.5 × 1.0 mm) was deposited by spray-coating method onto a porous FR4 laminate substrate using the procedure described in [20]. Before the P3HT deposition, the platinum interdigitated electrodes were deposited onto the substrate. The thickness of the RR-P3HT was not controlled during the deposition process. The approximate thickness was determined from AFM topography images.

### 2.3. Sensing Properties Measurements

**Figure 1:**
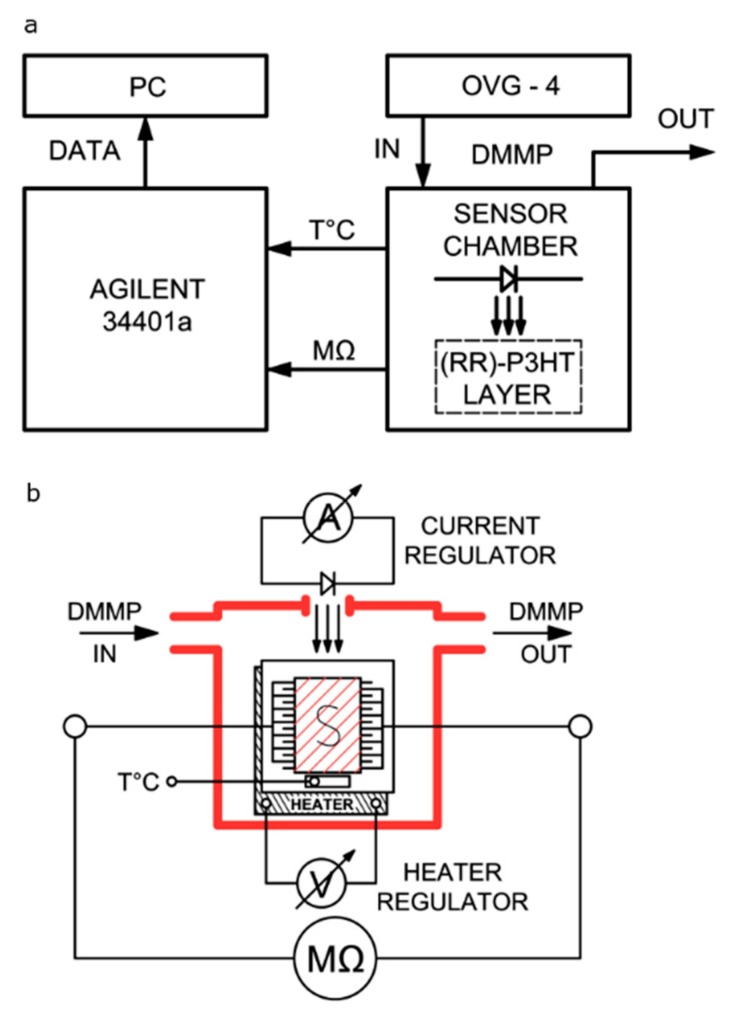
The sensing properties measurement set-up: (**a**) block scheme, (**b**) sensor chamber cross-section with a distance between the LED and the sample ≈ 5 mm.

Figure 1 shows the experimental set-up for the gas detection measurements. The fabricated sample was placed test chamber, which had a gas inlet and outlet, as well as an electrical feed-through. The chamber was designed specifically for this experiment. Its dimensions were as low as possible in order to place the sample inside the chamber while maintaining the limited volume for the flowing gas mixture (4 mL). DMMP, acetone and methanol vapours were produced using an Owlstone vapour generator (OVG-4) with certified permeation tubes. Synthetic dry air (≈5% relative humidity, Air Liquide) was used as a carrier gas. Resistance of the samples was monitored with a type 34401a Agilent Multimeter. The sample was activated by seven different light-emitting diodes: red (630 nm), amber (590 nm), yellow (575 nm), green (540 nm), blue (475 nm), magenta (650 nm) and white-neutral. The test chamber was designed in order to easily exchange the diodes, which were placed in the top of the chamber, just above the sample (1 cm). Figure 1 shows that the sensing structure was placed on a ceramic heater. Even so, the presented sensing responses were measured at room temperature. The temperature was monitored during the experiment by a Pt100 controller.

### 2.4. Topography Measurements

The film topography was imaged using an atomic-force microscope, AFM (XE-70, Park Systems Inc., Suwon, Korea), operating in non-contact mode in air. BS Tap300Al cantilevers were used. Images were processed with the Gwyddion2.45^®^ software [23] dedicated for SPM data isualization and analysis to correct sample inclinations and the influence of the z-scanning stage.

## 3. Results and Discussion

### 3.1. Sensing Properties

**Figure 2:**
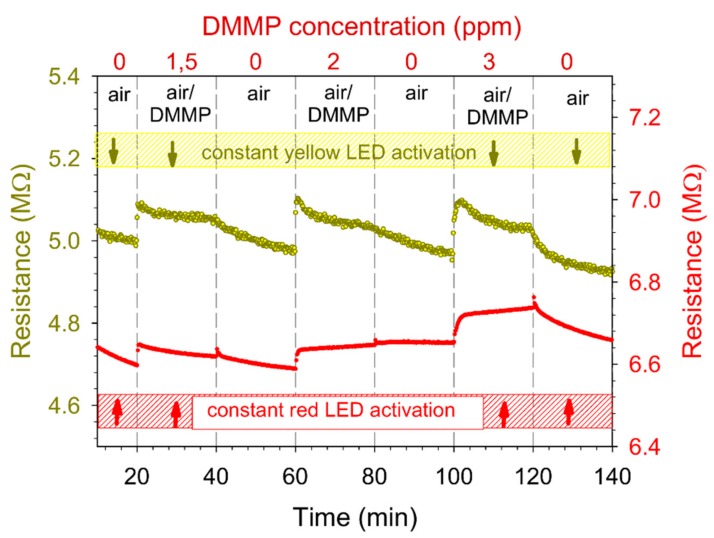
Time-dependent resistance plot in the room temperature of the RR-P3HT layer activated by red and yellow diodes in the presence of three different DMMP concentrations (1.5 ppm, 2 ppm and 3 ppm).

In our preliminary study [20], we reported the sensitivity of RR-P3HT to DMMP in room light conditions. In this work, we additionally activated it by using different LED illuminations, in order to enhance its sensing properties. We used seven different light-emitting diodes, including white. Figure 2 shows the sample time-dependent resistance plots for two of the LEDs—red and yellow. One can observe the dependence of the sample base resistance level on the irradiation wavelength (~5 for yellow light activation and ~6.5 M for red light). The values of the resistance for other LEDs with a wavelength between those of red and yellow range between 5 and 6.5 M. Conversely, the baseline dark resistance (without exposure to any light) of the sample was on the order of G to T. The discrepancy between these resistance values is indicative of the strong dependence of the conductivity of RR-P3HT on light activation.

**Figure 3:**
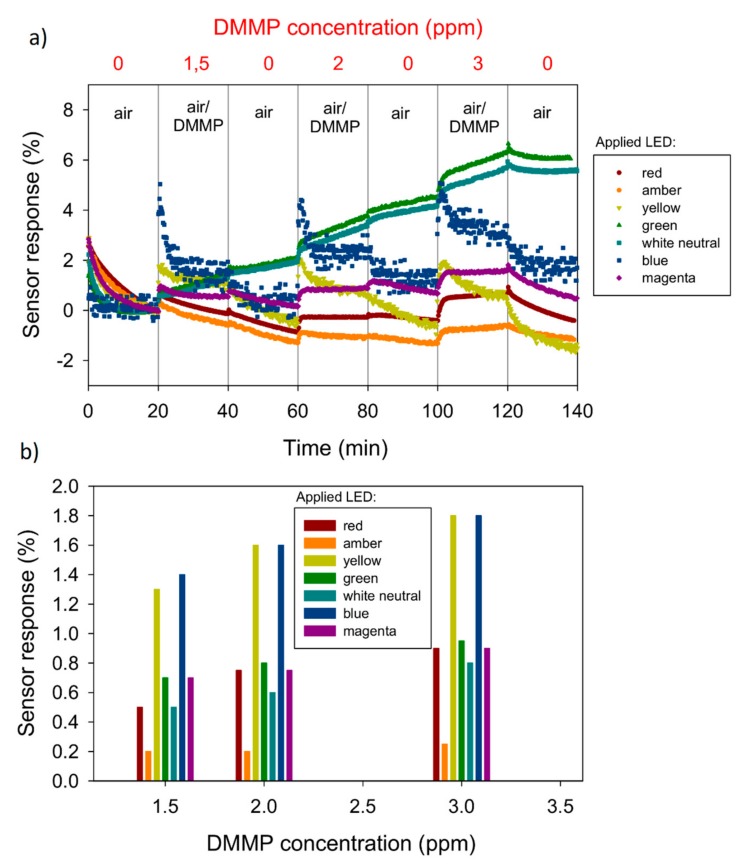
(**a**) The time-dependent sensor responses (SR) in the room temperature of the RR-P3HT layer activated by seven different diodes; (**b**) the dependence of sensor responses on DMMP concentration for all LEDs.

The sensor responses (SR) for all seven wavelengths are presented in Figure 3a. Figure 3b shows the dependence of the sensor responses on DMMP concentration for all LEDs. The concentration of DMMP was regulated by changing the DMMP/air mixture flow rate, according to the following formula:

(1) $$c=\frac{22.4q_{D}}{MQ},$$

where *c* is DMMP concentration (ppm), *q_D_*—permeation rate (747 ng/min), *M*—DMMP molar mass (*M* = 124.08 g/mol) and *Q* is a flow rate (mL/min). The sensor responses (SR) were determined from the time-dependent resistance plots. In order to quantitatively analyse the responses, the percent resistance change was calculated according to the following equation:

(2) $$SR=\frac{R_{0}-R_{gas}}{R_{0}}\cdot 100\%,\;,$$

where *R*_o_ is the resistance in air and *R_gas_* is the resistance of the sensor in the DMMP/air mixture. The air flow rate during regeneration cycles was constant (100 mL/min). The above figures show that the examined sensing material exhibits the highest sensitivity after activation by the blue (475 nm) and yellow (575 nm) light.

**Figure 4:**
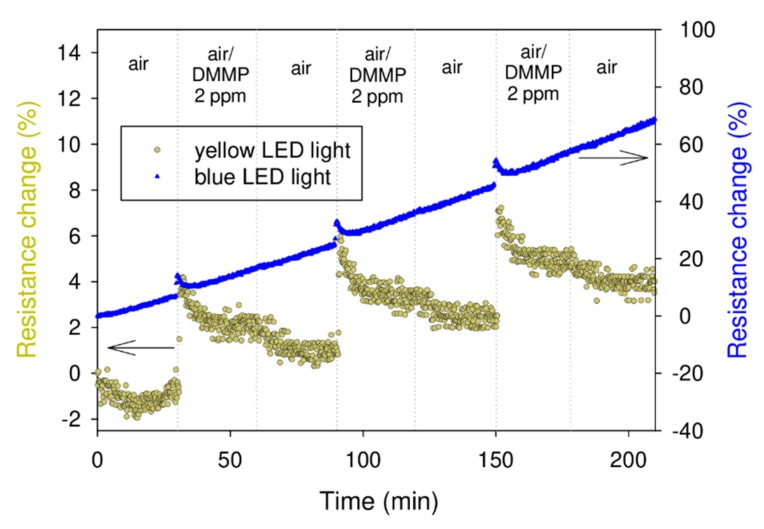
Electrical response of the RR-P3HT layer exposed to 2 ppm of DMMP under yellow and blue light illumination.

**Figure 5:**
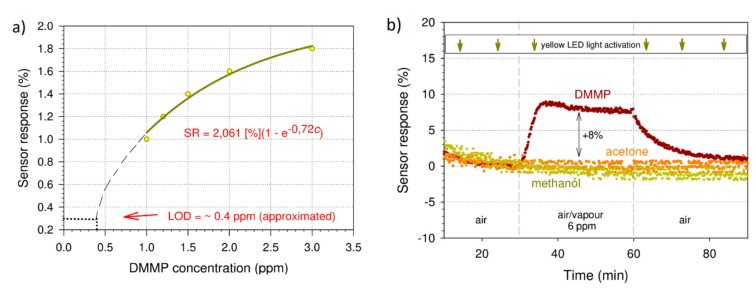
(**a**) The dependence of the sensor response on the DMMP concentration for the RR-P3HT layer activated by the yellow light; (**b**) the sensor response of RR-P3HT film exposed to the yellow LED light and 6 ppm of methanol (0% change), acetone (0% change) and DMMP (increase +8%).

In the next step, we studied the repeatability and short-term stability of the layers under exposure to the above light wavelengths (blue and yellow). The samples were exposed to 2 ppm of DMMP vapour in three cycles of absorption and regeneration (Figure 4). We can see that, in both cases (blue and yellow light activation), the sensing response was repeatable. There was, however, a high signal drift (0.332%/min) observed in the case of the layer that was exposed to the blue light. Conversely, for the layer irradiated with the yellow light, the drift was very slight (0.026%/min). We attribute the larger drift to the degeneration of the sensing layer, which was caused by the photons of higher energy (blue light). Although we did not investigate this degradation mechanism, it may be related to the light-induced doping of RR-P3HT with oxygen present in the air [24,25]. Therefore, we conducted further investigations with only yellow light activation. Figure 5a shows the sensor response as a function of DMMP concentration. This dependence was not linear. The limit of detection (LOD) was estimated from the interpolated curve. Considering 0.5% as the lowest detectable SR (the lowest value that was detectable above the noise for the studied structure, calculated as a standard variation in the signal), we obtained an LOD of 0.4 ppm. This value is lower than 1.1 ppm, which was obtained in the previous investigation [20] and is comparable with the LOD of the DMMP sensor based on poly(3-methyltiophene) which was reported by Ozturk et.al. [16]. However, it is still above the lethal concentration of sarin. Consequently, further sensor response enhancement is necessary. This is especially the case when comparing with the most recent study of a conjugated polymer-based electrical sensor aimed at detecting another nerve agent simulant, diethylchlorophosphate (DCP) [26] (5.88 ppb). Moreover, it must be noted that the obtained value is only estimated from the interpolated curve, since the lowest measured value was 1 ppm. The results of the selectivity studies are presented in Figure 5b. We did not observe any sensor response upon exposure to 6 ppm of acetone and methanol vapours, which are among the most common DMMP interferents. The studied concentration (6 ppm) was the highest concentration that could possibly be obtained in the applied gas dosing system for all three species (DMMP, acetone and methanol). However, this concentration can be considered only relatively high, especially when compared to the lethal sarin concentration. Thus, the selectivity of the investigated material in a concentration range of up to 6 ppm is acceptable. Another issue to address is the long-term stability of the sensing layer. As mentioned above, in Figure 4, one can observe the slight signal drift due to the material photodegradation. Although, for the yellow light activation, the signal drift is much less significant than for blue light, this should still be taken into account during the sensor device design.

### 3.2. Topography

**Figure 6:**
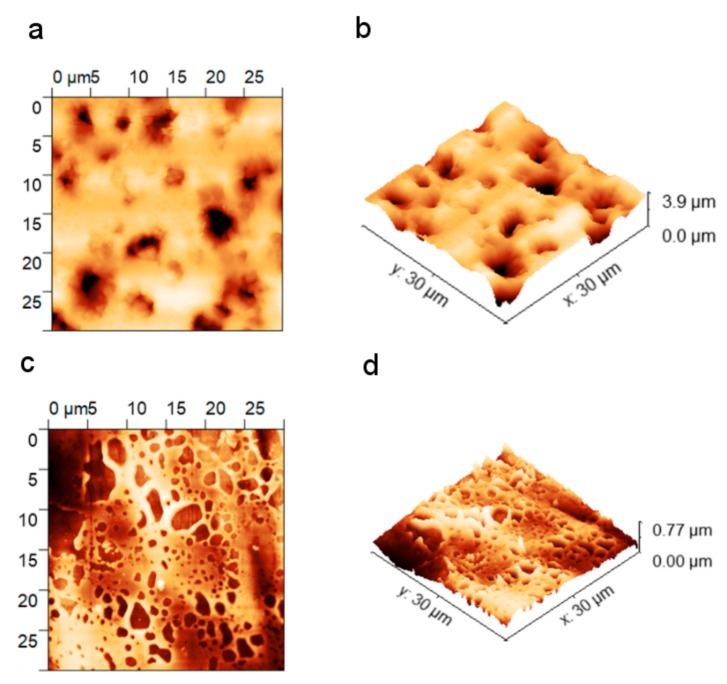
AFM images of the RR-P3HT sensing layer measured on a laminate: the polymer surface (**a**) and 3D-AFM view (**b**); and on metallization: the polymer surface (**c**) and 3D-AFM view (**d**).

The 30 × 30 μm^2^ AFM images of the sensing layer, after the investigation of the sensing properties with light activation, are shown in Figure 6. The polymer layer has a well-developed surface. The surface roughness, estimated by the root mean square (RMS), was equal to 474 nm. The layer thickness was not uniform—it varied from hundreds of nm up to a few m. This variation may be a result of the applied deposition technique, for which one can expect to obtain a thick film that is not equally distributed on the substrate. However, the advantage of the spray-coating method is its simplicity. The well-developed surface and high roughness are considered advantageous in gas sensing: more active adsorption sites are available for a more developed a sensing layer surface. The topography image revealed the degradation of the polymer layer, which was probably caused by light illumination. The layer is discontinuous and riddled with micron holes which may be a result of the photodegradation of the sensing layer. This is in accordance with our observations from the gas sensing experiments, where we observed an increase in the electrical resistance of the sample, which was caused by light irradiation (especially for the blue LED). The RMS parameter of roughness determined on the Pt electrodes was equal to 85 nm. This shows that the porosity of the substrate significantly increases the roughness of the polymer layer. However, this study was restricted to only the one substrate. In order to investigate the influence of the porosity of the substrate on the sensing performance, a detailed study comparing the sensing responses of P3HT layers deposited on substrates with different porosities would be required.

### 3.3. Sensing Mechanism

Polythiophenes, including P3HT, have a Lewis basic nature, due to the free electron pairs on their sulfur atoms [27] and are hypothesised to form adducts with Lewis acids. Such interactions are well-reported in the literature, particularly with boron trifluoride, which is commonly used both to facilitate polymerisation of thiophene-based monomers and to improve the conductivity of thiophene-bearing conjugated polymers [28]. The aforementioned improvement in conductivity stems from doping-induced charge carriers being produced, due to the altered electron density distribution across the P3HT chain, caused by interactions with Lewis acids.

Conversely, virtually no works are reported on the doping of polythiophenes with Lewis bases. This is expected, as any interactions between P3HT and another Lewis base should adversely affect the properties of P3HT, particularly its conductivity.

The DMMP molecule has a Lewis base nature [29], therefore the loss of P3HT conductivity upon exposure to it can be considered in line with the above hypothesis. Even so, an in-depth experimental investigation would be required to confirm its veracity.

Another possible mechanism which explains the declining P3HT conductivity upon exposure to DMMP may be based on an increase in inter-chain distance, caused by the incorporation of DMMP molecules into the RR-P3HT matrix, which affects the electron hopping between RR-P3HT chains. This is in line with the results obtained by Wang et al. [30], who postulate that DMMP induces conformational changes in polythiophene chains, which likely also significantly contribute to the formation and decay of conductive paths in the film, directly effecting changes in its macroscopic conductivity.

The above-described mechanisms may be enhanced by light illumination, however it is unclear whether light illumination only increases the baseline conductivity of P3HT (e.g., via photogeneration of charge carriers), making the losses of conductivity caused by the interactions with DMMP appear more significant, or whether it also affects the interactions between P3HT and DMMP.

## 4. Conclusions

In summary, we investigated the RR-P3HT photoconductive polymer—deposited by the spray-coating method—as a potential material for chemo-resistive sensors of the sarin simulant, DMMP. In order to enhance the sensing properties of RR-P3HT, we applied activation by light-emitting diodes with various wavelengths. The study was conducted at room temperature, using RR-P3HT films deposited on the highly porous FR-4 laminate. The highest responses to DMMP vapours were observed under the blue (475 nm) and yellow (575 nm) light excitation. The layer exposed to the blue light turned out to be less stable, which we believe was a result of the material photodegeneration. Applying the yellow diode activation, we obtained an estimated limit of detection of 0.4 ppm. The electrical responses of RR-P3HT to other organic vapours, like acetone and methanol, were also studied, and the material was found to be highly selective. The future work will be focused on the further improvement of detection limit and stability. The main issue that should be addressed in the next work is the photodegradation and its influence on the sensor performance. A detailed study of sensing mechanisms for light-activated layers has to be performed. Another open question is the influence of the film porosity on the sensing properties. In order to perform these studies, another deposition technique should be applied to better control the film thickness and its uniformity.
